# Comparative Study on the Cooling Effects of Green Space Patterns in Waterfront Build-Up Blocks: An Experience from Shanghai

**DOI:** 10.3390/ijerph17228684

**Published:** 2020-11-23

**Authors:** Yunfang Jiang, Shidan Jiang, Tiemao Shi

**Affiliations:** 1 Center for Modern Chinese City Studies, School of Urban and Regional Science, East China Normal University, Shanghai 200062, China; 51183902011@stu.ecnu.edu.cn; 2 Institute of Eco-Chongming, Shanghai 202162, China; 3 Institute of Spatial Planning and Design, Shenyang Jianzhu University, Shenyang 110168, China; tiemaos@sjzu.edu.cn

**Keywords:** green space network, green space pattern, spatial morphology, cooling effect, ENVI-met simulation, boosted regression trees (BRT), marginal effect (ME), Shanghai

## Abstract

Different structural patterns of waterfront green space networks in built-up areas have different synergistic cooling characteristics in cities. This study’s aim is to determine what kinds of spatial structures and morphologies of waterfront green spaces offer a good cooling effect, combined with three different typical patterns in Shanghai. A multidimensional spatial influence variable system based on the cooling effect was constructed to describe the spatial structural and morphological factors of the green space network. The ENVI-met 4.3 software, developed by Michael Bruse at Bochum, German, was used to simulate the microclimate distribution data, combined with the boosted regression tree (BRT) model and the correlation analysis method. The results showed that at the network level, the distance from the water body and the connectivity of green space had a stronger cooling correlation. The orientation of green corridors consistent with a summer monsoon had larger cooling effect ranges. In terms of spatial morphology, the vegetation sky view factor (SVF) and Vegetation Surface Albedo (VS_Albedo_) had an important correlation with air temperature (T), and the green corridor with a 20–25 m width had the largest marginal effect on cooling. These results will provide useful guidance for urban climate adaptive planning and design.

## 1. Introduction

The ecological functions of urban blue-green spaces have become a popular topic in geography, urban planning, environmental science, and urban ecology in recent years [1,2,3,4]. The cooling effect of urban blue-green spaces is a very significant function and plays a critical role in mitigating urban heat islands (UHIs) [5,6,7]. As a special ecological landscape composed of water bodies and green spaces, waterfront green spaces have become important urban areas that provide a liveable ecology and microclimate environment for citizens. A growing number of studies have shown that blue–green spaces can significantly improve urban ecological problems and microclimate effects and increase human comfort perception [1,8,9]

The cooling effect of urban blue–green spaces, also known as urban cool islands (UCIs), is an important ecological effect that has attracted increasing attention [6,7,8,9,10]. Waterbodies include rivers, lakes, and reservoirs, while the green spaces consist of trees, shrubs, and grass, which make up the blue–green spaces in urban areas. The cooling mechanisms of such spaces are also different. Water bodies have the physical characteristics of a high specific heat capacity and low thermal conductivity and cool by evaporation, which forms a “thermostatic effect” [11]. Green spaces achieve cooling by regulating the surface heat exchange, mainly through the transpiration and shadowing effects of urban vegetation and the selective absorption and reflection of solar radiation [12,13,14].

With respect to the cooling effect of green space, more attention has been paid to the single composition and morphological factors. For green areas, generally, large green spaces can form a relatively stable microclimate, and their cooling effects can be more significant and positive. The landscape shape index (LSI) of green space is also an important factor impacting the cooling effect, and the cooling intensity of small-sized green spaces with complex shapes can be limited or even have a negative effect [10,15,16,17,18]. In a case study of Fuzhou city, circular green space had higher heat dissipation efficiency than square green space [17]. The vegetation configuration of green space also has a significant effect on the cooling effect. The cooling effect of green spaces with trees was shown to be stronger than that of grass, and the cooling distance was significantly related to the height of the tree canopy in an empirical study of London [16]. In addition, the landscape composition and distribution of green space can significantly impact the cooling effect [5].

The cooling effects of water bodies are mainly affected by the size, shape, location, wind direction, and surrounding landscape pattern [19,20]. Quantitative studies have shown a significant positive correlation between the water area and cooling intensity [21]. Water bodies in the upwind direction have the best effect on increasing temperature and reducing humidity [22]. The waterfront space promotes air circulation due to its openness, and the dominant wind direction further enhances the water-cooling effect [16]. The average cooling distance and the amplitude of the temperature drops of water bodies are 740 m and 3.32 °C, respectively [19].

The synergetic cooling effect of green space and water bodies has been a concern in recent years. Vegetation can affect the radiation balance of water bodies, adjust water temperature, and promote air convection through shading, indicating that high-density vegetation can reduce the coastal water temperature [23]. The cooling effect under a combination of water bodies and vegetation is significantly better than that without vegetation and water bodies [24,25]. The landscape composition was considered as an important factor influencing the cooling effect of blue-green space [26,27]. Landscape configuration (pattern and shape), rather than composition, is a more important factor influencing the cooling effect of blue-green space [5,26,27,28,29]. These studies have primarily focused on a discrete patch within the landscape matrix and identified the cooling effects as being dependent on the sizes and shapes of blue-green space [26,28].

Although a large number of studies have been carried out on the cooling effect of urban blue-green space, as well as many quantitative studies on the relationship between the spatial morphology factor of green space and its cooling effect, many mechanisms of the impact factors still require further study. Against this background, this study is focused on the local thermal environment in a hot-humid climate by computational dynamics simulation. This study comprises (1) quantification of the SCEs of urban blue and green spaces for mitigating summer UHIs due to different green spatial combinations in build-up area; (2) utilization of a validated ENVI-met model to qualify the effect of different spatial morphology variables of blue-green space fabric, which belongs to three typical green patterns in waterfront area on a local medium-size scale. (3) From a holistic perspective, this paper applies boosted regression trees and the geographic information system method to further study the influence of different structural and morphological factors of the green space network in the waterfront area on air temperature change.

This study’s aim is to (1) determine what kinds of spatial structures and the morphologies of waterfront green spaces offer good cooling effect, combined with comparative experience in the urban building-up area, and (2) develop research method on multivariate contribution analysis of the cooling impact factors in typical urban waterfront green space. The study tasks were addressed by co-modeling the thermal benefits of blue-green space design with urban ecological space dynamic collaborative development. The outcomes of this study will serve as green patterns for adaptive UHI mitigation in waterfront area on a local medium-size scale and contribute to further improving thermal comfort for urban residents.

## 2. Study Area and Methodology

### 2.1. Methodological Framework

**Figure 1:**
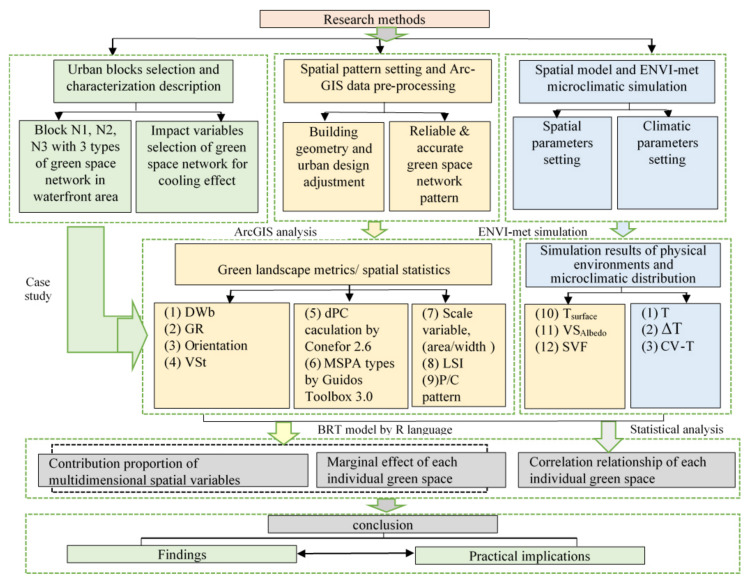
Flow chart of the overall study. DWb: Distance from water body; GR: Green space ratio; VSt: Vegetation structure; dPC: The decrease probability connectivity; MSPA: Morphological spatial pattern analysis; LSI: Landscape Shape Index; P/C pattern: Patch/Corridor pattern; T_surface_: Surface temperature (°C); VS_Albedo_: Vegetation surface albedo; SVF: Sky view factor; T: Air temperature (°C); ΔT: The difference between T and the mean T of the entire study area; CV-T: Coefficient of variation of air temperature.

The methodological framework of this study is shown in Figure 1. The study area contained 3 cases with different types of green space network patterns in waterfront urban blocks. The typical process for cooling effect studies involves four major steps: (1) Urban block selection and characterization descriptions to quantify the spatial structure and morphology of the green space. The spatial patterns were chosen based on satellite data combined with regulatory urban design data to identify the distribution of green space. (2) Spatial pattern extraction, urban design adjustment and spatial analysis. Using the software platforms ArcGIS (Esri, Redlands, California, USA), Guidos Toolbox (European Commission Joint Research Centre (JRC), Ispra, Italy), and Conefer 2.6 (by Santiago Saura and Josep Torné, at the Polytechnic University of Madrid and the University of Lleida, Spain), the climate-related spatial description variables were calculated from the network structure level to the individual spatial morphology factors (attribution data, spatial analysis, proximity analysis, and patch analysis), as well as to calculate the landscape indicators. (3) Three-dimensional spatial model construction using the ENVI-met software (developed by Michael Bruse, at the Bochum, German) and a microclimatic simulation. The simulation results provided the physical spatial variables (Tsurface, Albedo, and Sky view factor (SVF)) and microclimate index values (T, ΔT, and CV-T). (4) BRT modelling analysis and statistical analysis of the results. Correlation determinations between the cooling effect and different types of landscapes were processed to obtain the spatial structure and morphological factors’ impacts on the cooling effect, as well as validate the related threshold value (Figure 1).

### 2.2. Study Area

**Figure 2:**
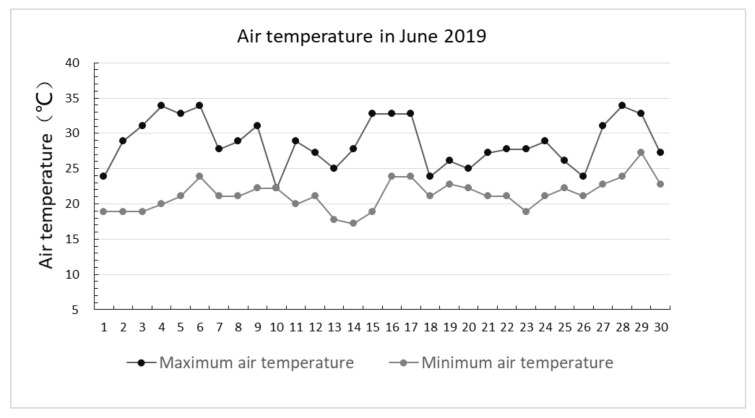
T (°C) in June 2019 in Shanghai.

Shanghai is located at the north latitude of 31°12′ and the east longitude of 121°30′ and is situated in the plain area of East China in the Yangtze River delta. Shanghai enjoys a subtropical monsoon climate, four distinct seasons, high temperature, and abundant rainfall in summer and low temperature and less rainfall in winter. With global warming and climate change, extreme weather phenomena have increased for many years in Shanghai City, particularly extreme weather such as high temperature heat waves and heavy summertime rains. In recent years, according to meteorological data of the Chinese Meteorological Administration Centre, extreme weather phenomena have been increasing in Shanghai, particularly high temperatures and heat waves. Shanghai is a typical subtropical monsoon characterized by high temperatures in June (Figure 2) and abundant rainfall in the summer.

**Figure 3:**
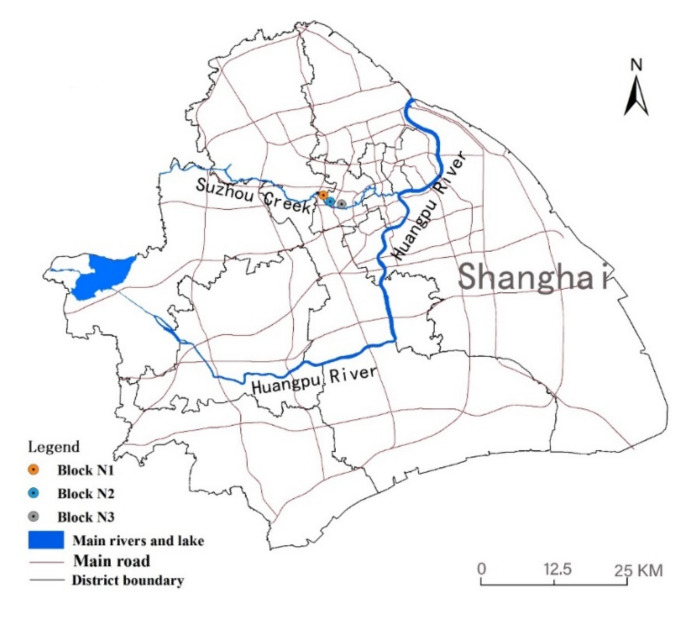
Locations of the study areas in Shanghai city.

**Figure 4:**
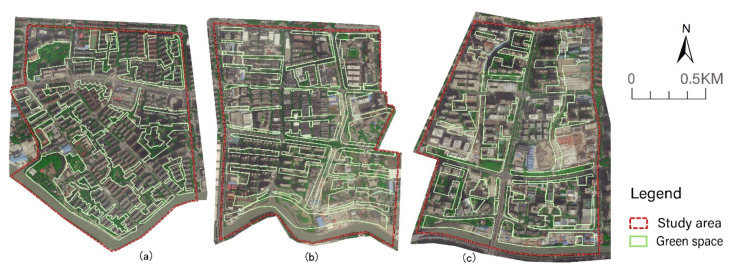
Satellite image and the green space network of three study areas in 2019 ((**a**) Block N1; (**b**) Block N2, (**c**) Block N3).

The Suzhou Creek Corridor is an important river that runs through the Shanghai metropolis and spans many districts from west to east. The land use of the waterfront area is diverse, and the riparian green space in most sections of the creek is open and continuous. Based on the abovementioned large climate background, three study areas on the south bank of Suzhou Creek in the city centre were selected and were all located in the upwind direction (Figure 3). The width of the water body was approximately 56 m, and the areas of the three blocks were similar. The three blocks have great differences in the spatial patterns of their green space networks (Figure 4).

Block N1 covers an area of 1.44 km^2^, with a green space ratio of 28.48%. The main orientation of the green space corridor is SE–NW as the vertical direction, which extends to both sides to form the horizontal branches of the green space, forming a grid pattern in the green space network. The green space network in this area is more evenly distributed and relatively dense. The width of the riparian green space is narrow and only 35 m wide. In the south-east section of the riparian area, there is a large green space approximately 95 m wide.

Block N2 covers an area of 1.38 km^2^, with a green space ratio of 28.37%. The green space in this area is mainly west-east oriented, parallel to the water body of the creek. There is only one S–N green corridor along both sides of the inner road. The southern riparian area has a continuous, wide green corridor from west to east, with a width of approximately 90 m.

Block N3 covers an area of 1.34 km^2^, with a green space ratio of 27.86%. The green space layout of this area is mainly wide S–N green corridors, with a small number of different-sized green patches and narrow green branches. The continuity of the riparian green space is slightly lower than that of block N2, as the wide riparian green spaces are divided into two parts by the road, and a building area was built in the middle of the eastern riparian green belt.

### 2.3. Spatial Description Variables of the Waterfront Green Space Network

#### 2.3.1. Multidimensional Spatial Variables Framework

To identify the best mitigation UHI solutions adapted to a particular building environment, the efficiency of each mitigation solution may be tested running micro-climate models on a limited number of built environments. A given set of climate-related parameters such as aspect ratio, albedo, or vegetation density were performed as the conceivable solution for defining the geometric and land-cover properties for each city [30].

The spatial configuration of system properties is much more difficult to quantify. Attempts have focused on describing the spatial characteristics of the spatial relationships among the landscapes’ constituent patches [5,31,32]. The green spaces connected with waterbodies intensified the cooling effects [19]. Modelling or proposing the optimized spatial arrangement has identified and affected the cooling effects [26]. Planting smaller networks of vegetation could be a significant part of the mitigation of UHI intensity [33]. Water feature corridors have a certain influence on a certain range of downwind zones; and the different orientation of green corridors and green patches made different contributions to the cooling effect [34]. The morphological spatial pattern analysis (MSPA) mode was used to identify the main types and structure of green spaces in the landscape, which will make the foundation of the green space network [35,36,37].

Meanwhile, the spatial morphological variables of each green space were used to describe the spatial relationship for UCIs. Sky view factor (SVF), one of the geometric properties, defines the ratio of sky hemisphere visible from the ground and informs about the microscale relationships between groups of factors, such as the sizes, types, and locations of green space parcels which together influence the effectiveness in modifying the local microclimate [30]. Related studies also pointed out that the cooling effect may be affected by the character of the surrounding environment of blue–green space (e.g., the land use and land cover pattern and the height/density of the surrounding buildings) [6,29].

The variables of holistic structure of the waterfront green space network, in the study, were included the green space’s orientation, distance from water, and connectivity degree (here adapted as decreased probability connectivity, abbreviated as dPC [38,39,40]); the types of morphological spatial patterns (MSPA types); the green space ratio; and the vegetation structure. The spatial morphology variables of the specific green space were used to describe the three-dimensional morphological characteristics of each specific green space, including the green space area, the GWd, the LSI, the VS_Albedo_, SVF, and the T_surface_.

At the specific urban meso-scale of the waterfront area, we selected the climate-related variable system. The selection of the multidimensional spatial variables system for meso-scale region is determined based on the previous study; thus, it is suitable for the research of spatial microclimate characteristics in the specific scale in different city locations. Due to the detailed scrutiny of different multidimensional spatial variables for waterfront green space, the values assignment would have undergone a corresponding change for describing the different cases in specific location, and the obtained conclusions might reflect the main impact of variable performance on urban design. The multidimensional spatial variables were selected in Table 1.

#### 2.3.2. Control Variables

Additional control variables were derived to account for factors previously hypothesized to influence the thermal effect, such as size, density, and climatological conditions [51]. To study the differences in the cooling influence from the network structure of the green space and the cooling source’s (water body) connection to the introduction and exchange of cold air in the riparian area in each block, in the stage of the schematic design, the two influencing factors, namely, the green space ratio and vegetation structure, were preset with the same fixed proportion values as the control; thus, the microclimate effect of vegetation’s quantitative factors was excluded from the correlation analysis in the complex variables system. The goal setting mainly involved studying the cooling impact changes caused by the multidimensional morphological and structural differences of the waterfront green space.

#### 2.3.3. Quantifying the Spatial Configurations Variables

##### The Network Structure Variables

The data management tools in ArcGIS 10.4 were used to extract the geometric centre coordinate points of each green space via the tools for feature to point, and then the nearest analysis in the analysis tools was used to calculate the shortest distance from the geometric centre of each green space to the water body bank.

The decrease in probability of connectivity (dPC) was selected to evaluate the connectivity degree of the green space. The definition of the dPC is important for a green patch to maintain the overall habitat availability in the landscape [52,53,54]. The contribution of each green space in the connectivity to the overall green space network was evaluated using the dPC values. To calculate the dPC values, first, the probability of connectivity (PC) was calculated, and then, the dPC values were calculated based on the PC. The calculation formula is expressed in Formulas (1) and (2):

(1) $$\mathrm{PC}=\frac{\sum_{i=1}^{n}\sum_{j=1}^{n}a_{i}a_{j}P_{\mathrm{ij}}}{A_{L}^{2}}$$

where n represents the total number of patches in the landscape, and P_ij_^*^ is the maximum product probability of all of the possible paths between patches i and j (including direct dispersal between the two patches). a_i_ and a_j_ are the areas of the habitat at patches i and j, and A_L_ is the total landscape area. The PC values are bounded (ranging from 0 to 1) and defined as a probability of coincidence in a similar way to the degree of coherence provided by Jaeger et al., 2000 [55]:

(2) $${\mathrm{dPC}}_{k}=\frac{\mathrm{PC}-{\mathrm{PC}}_{\mathrm{remove},k}}{\mathrm{PC}}\times 100\%$$

where PC_remove,k_ is the overall possible connectivity of the remaining patch after the removal of the k’th green patch. It should be noted that PC was conceived for use as a relative metric to evaluate the importance of landscape elements or changes [39,52]. Based on relevant studies [38], the threshold distance of the connectivity was set as 50, and the probability of the connectivity of patches was set as 0.5. Then, the Conefor Sensinode 2.6 software (by Santiago Saura and Josep Torné, at the Polytechnic University of Madrid and the University of Lleida, Spain) was used to calculate the dPC value of each green space.

Morphological spatial pattern analysis (MSPA), an image-processing method based on concepts from mathematical morphology [56,57], can reflect the structural links between core patches. Notably, this feature cannot be achieved using other methodologies, such as structural indices or theoretical graph approaches [58]. To partition the MSPA types of green space, Guidos Toolbox 3.0 (European Commission Joint Research Centre (JRC), Ispra, Italy) was used for the morphological analysis. The accuracy of the binary TIFF format raster data was 0.2 m × 0.2 m, and the edge width was set to 50 mm. In this way, the morphology classification of the green space network based on MSPA analysis was determined.

**Figure 5:**
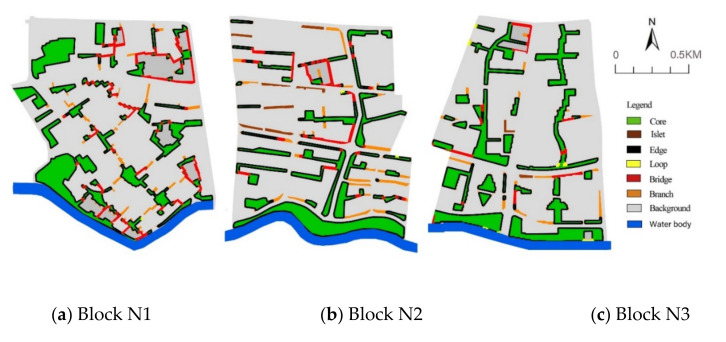
Morphological analysis of the three built-up areas.

Green space coverage areas were divided into seven categories: core, bridge, loop, branch, edge, and islet (Figure 5). For quantification the MPSA type related to UCIs, the core area was further divided into core patches and core corridors to distinguish the differences in cooling effects with different shape features. The loop area was very small, so it was not analysed in the regression analysis. As a classification variable, according to the importance of the connectivity of the green space, eventually the values of different MSPA types were assigned as follows: The value of the branch type was 1; the value of the island type was 2; the value of the core patch type was 3; the value of the bridge type was 4; and the value of the core corridor was 5.

##### The Morphological Factors at the Microlevel

The green space landscape pattern was used to describe the characteristics of each green space, i.e., the patches or corridors, solely for comparing the effects of the landscape types on the cooling effect. This variable was also set for the classification variable: The value of the patch pattern was 1; the value of the corridor pattern was 2; and the width and area of the green space patch and corridor were continuous variables describing the spatial scale, whose values were confirmed by the ArcGIS measurement of the actual surface area of the green space and the cross-sectional width, which was perpendicular to the green corridor’s ventilation direction.

The variable of the LSI was used to describe the complexity of the landscape patterns by calculating the degree of deviation between the shape of a plaque and its square of the same area. It is generally believed that the edge effect of complex patches has a great influence on the microclimate of the region. The LSI value of each green space was calculated via patch analysis in ArcGIS 10.4; the specific calculation is expressed via Formula (3)

(3) $$\mathrm{LSI}=\frac{0.25L}{\sqrt{A}}$$

where LSI is the landscape shape index, L is the perimeter of the green space, and A is the area of the green space.

The values of width from ArcGIS measured the cross-sectional width perpendicular to the green corridor’s ventilation direction. The three-dimensional morphological variable SVF was used to describe the degree to which the sky was obscured by buildings, vegetation, etc. This variable reflected the effect of vegetation cover shading and longwave radiation on improving the thermal environment. The variable of VS_Albdo_ represented the three-dimensional morphological influence of green space vegetation and the ambient factors, resulting in different absorption and reflection values of solar radiation on the surface of the green space, which varied the distribution of T. The values of the three indices were obtained from the surface data through the ENVI-met 4.4 simulation results.

### 2.4. Simulation Model and Scheme Design

#### 2.4.1. Urban Design Methods for the Green Space Network

The scope of this study area is the urban mesoscale, from approximately more than 1 km^2^ to a pre-defined extent. Therefore, this research was designed to determine the spatial interactions between the network patterns of green space and the microclimate thermal environment. The experimental path attempted to reduce the interference of the external spatial fabric caused by different building enclosures, so the urban design control method with a simplified structural green network pattern as the dominant spatial pattern was adopted.

**Figure 6:**
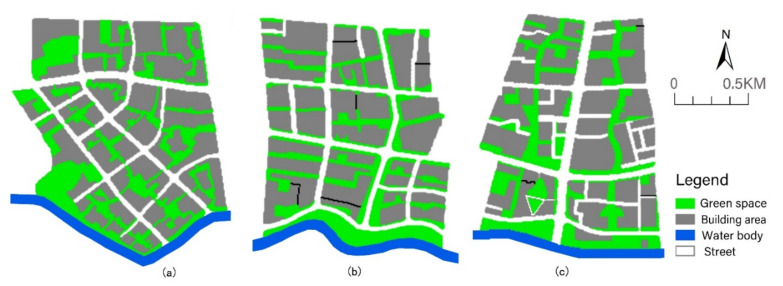
Modelling diagram of the three built-up areas (from left to right): (**a**) Block No. 1 (N1); (**b**) Block No. 2 (N2); (**c**) Block No. 3 (N3).

In this study, the structural green space pattern was extracted from high-definition aerial photography using GIS tools. In each block, the other space was partitioned by the green space network, which was taken as the whole built-up group area, eventually forming the structural green space pattern surrounded by the building space. This simplified simulation model under the control method of urban design can reduce the disturbances from other external details of open space impacts on the microclimatic environment, thus highlighting the single cooling effect of a typical green space network structure. The three models formed by abstraction represent three typical network patterns of waterfront green space near Suzhou Creek. These three models were used as the research cases in this paper (Figure 6).

#### 2.4.2. ENVI-Met Dynamic Simulation Method

Numerical models are widely used in urban microclimate research [59]. ENVI-met is one of the most common computing programs and was developed by Bruse and Fleer in Germany to simulate microclimate interactions between the urban surface, vegetation, and atmosphere at both the meso- and microscopic scales [60]. This study was simulated via ENVI-met 4.4.3, which was accessed on 24 June 2019. The weather data in this study were obtained from the Weather Underground website and the weather station of Shanghai Hongqiao Airport [61]. The initial input weather parameters are shown in Table 2, and the T values and humidity were the average values for the whole day of 24 June 2019.

To compare the effect of the structural green space on the microclimate, the settings of each spatial grid and parameters were the same as the control variables used for the similar three-dimensional green space configuration. Namely, each grid was distributed using a two-layer structure of plant covers with trees and grasses, composed of a 5 m high and 5 m wide tree canopy and 0.5 m of high grass. Since the tree spacing was 6 m, the length and width of the grid cell in the horizontal direction was set as 6 m × 6 m. In the vertical direction, each grid was set to the height of one floor—that is, the height was set to 3 m/grid cell. In terms of materials, this article studied the form of green space. To attenuate the influence of street material on the cooling effect of green space, the street material was set to loamy soil, and the materials of the nesting grids were also loamy soil. Table 3 shows the detailed spatial parameter settings. The simulation model based on raster images of the study areas showed the spatial composition of the two-dimensional land use, as illustrated in Figure 6.

### 2.5. Statistical Methods

#### 2.5.1. The Holistic Thermal Environment Status Analysis

To analyse the thermal environmental impact of the holistic green space network, the cooling difference data corresponding to the three green space network patterns were compared. By calculating the air temperature difference (ΔT) of the green spaces in the three study areas to analyse the microclimate status of diurnal and nocturnal change, we directly determined the overall cooling effect of the three study areas.

In the analysis of the corresponding relationship between the T factors in the green space network, the coefficient of variation (CV) data of T was used to analyse the fluctuation of the microclimate variables of the green space network system with different green spatial patterns in three blocks. The calculation formula is expressed as Formula (4):

(4) $$\mathrm{CV}=\frac{\mathrm{SD}}{\mathrm{MN}}$$

where CV is the coefficient of variation, SD is the standard deviation, and MN is the average of each index.

#### 2.5.2. The Contribution Proportion of Multidimensional Spatial Variables to the Cooling Effect

The growth regression tree model (boosted regression trees, BRT) was developed by Elith et al. [62]. First presented in 2008, this model is a method of machine learning that combines the advantages of regression models and growth models to address the interactions between nonlinear variables and variables and is significantly different from traditional linear regression models [62]. In addition, the BRT model can simulate the MEs of arguments. In a traditional regression model, the ME of the arguments is a constant that cannot capture the complex and nonlinear relationships of the variables. The BRT process of fitting multiple trees minimizes the weak predictive power of a single tree model. Therefore, this model has greater predictive power than most traditional model methods. BRT models are widely used in species distribution [63], urban heat island effects [42], soil carbon concentration [64], cold air paths [65], etc. This method is currently being explored and applied in urban microclimate research.

The BRT model was used to explore the contributions of multidimensional spatial indices of green space composition and morphology to the thermal environment. We used the dismo package in the R language to implement the growth regression tree model [60]. The causal variable was the simulated air temperature of the green space, and the independent variables were the descriptive spatial variables, i.e., the DWb, the MSPA type, the connectivity degree, and the other network-level description variables and spatial morphology variables. The model parameters were set as follows: The decision tree complexity was 5, the learning rate was 0.01, the split ratio was 0.5, and the data type was Gaussian, based on previous research [42,65]. This model extracted 50% of the data points for analysis at a time, with 50% for training and 10-fold cross-validation used to estimate the number of optimal trees.

#### 2.5.3. Marginal Effect and Correlation Analysis of Individual Spatial Factors to Cool Effect

To analyse the thermal environment correlation of the green space network, the paper includes an analysis of the cooling effect at the urban mesoscale of the network structure factors and the cooling influence of the spatial morphology factors. The influencing factors at the network level of the green space were analysed as the distance from the water body, the DPC, the green space orientation, and the MSPA types.

The correlation analysis also included the spatial morphology factors impacting the cooling effect. The factors at the microscale level of green space were VS_Albedo_, Tsurface, SVF, and the scale and width of the green space.

The cooling correlation of the continuous variable, namely, the distance from the water body; the dPC values; and the values of VS_Albedo_, T_surface_, SVF, and GWd, was analysed through the ME changes of each variable based on the BRT model, and the threshold value of the cooling effect from each influenced factor was also preliminarily analysed. Based on the statistical classification method, the data of each morphological factor were classified by their general grade values based on existing research. The relationships between the specific morphological variables of different grades of green space and the T values was further analysed, and the direct cooling effect of each morphological factor was discussed in detail.

## 3. Results

### 3.1. Component Contribution Analysis on Cool Islands from the Waterfront Green Space Network

#### 3.1.1. Characteristic Analysis of the Cool Island for the Holistic Network

**Figure 7:**
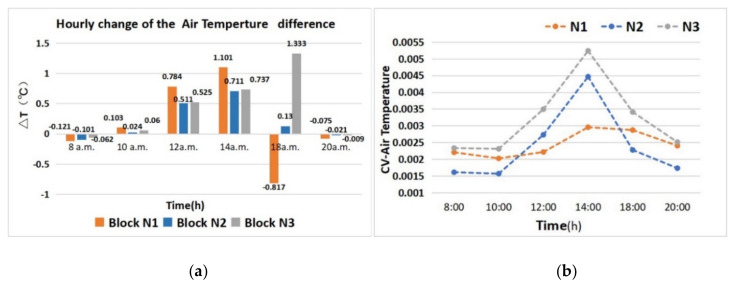
Analysis of the variation characteristics of the air temperature of the three green space network patterns during different hours. (**a**) ΔT comparisons; (**b**) the CV-T values comparisons.

The cooling effects of the three studied green space network patterns are obviously different (Figure 7a). In the diurnal period, the ΔT values of the green space from block N1 showed that the cooling effect was significant. At 12 a.m. and 2 p.m., the ΔT values reached 0.784 °C and 1.101 °C; the cooling effect of block N3 was lower than that of N1, and for block N2, the ΔT values were 0.525 °C and 0.737 °C at 12 a.m. and 2 p.m. In the nocturnal period, the ΔT values of block N1 were lower than those of block N3, which featured the best cooling effects of the three blocks. According to the variation of ΔT at different times, we found that the network interweaving pattern of green space in block N1 achieved the best cool island state. The cooling range of the green space network dominated by the S–N green corridors experienced significant changes over the whole day.

Based on an analysis of the CV value changes of the three green space networks (Figure 7b), the T value fluctuation in the green space of block N1 was generally the lowest. The T value inside the green space of block N2 changed greatly, which was consistent with the rise and fall of the solar altitude angle in the diurnal period. The fluctuation of the T value in the green space of block N3 was the largest, which was consistent with the change characteristics of block N2. Further, the fluctuations of the internal T were significantly greater than those of block N2.

The cool island effect of block N1 was relatively stable. To some extent, the green space network with good connectivity indicated only a small internal cool island difference. The cool island effects of block N3 and block N2 were very different, and generally speaking, the biggest difference between them was related to the openness of the green corridors. The openness of the green corridors was directly related to the orientation and width factors. The corridor orientation in the S–N direction was more consistent with the wind direction; the corridor orientation in the east–west direction obstructed the introduction of the southeast monsoon during the summer to an extent, and the S–N orientation corridor helped lead the cold air from the water body in the south to the internal green space. In terms of width, block N3 also featured a wider green corridor. As a result, the internal spatial change of green space in block N3 was significantly greater than that in block N2.

#### 3.1.2. Relative Importance of Spatial Variables of Green Space to the Cooling Effect

The contribution ratios of the spatial index factors of the three green spatial patterns to the cooling effect were different. Through a BRT regression analysis between the established morphological index of the waterfront green space network and the T values, the importance of the cooling effect of spatial indices describing the green space morphological factors in different waterfront blocks was obtained (Table 4). In block N1, SVF (22.18%), VS_Albedo_ (21.89%), DWb (18.11%), and GWd (10.67%) were the most important influencing variables. In block N2, the main influencing variables were dPC (19.34%), T_surface_ (18.45%), VS_Albedo_ (16.72%), and DWb (10.79%). In block N3, the most important influencing variables were SVF (22.69%), distance from water (14.78%), dPC (14.52%), and VS_Albedo_ (10%). The contribution ratio of the other variables ranged from 0.06% to 10.53%, all with low relative importance. According to the variable classification, the cool island effect of green space in the three blocks was obviously closely related to the distance from the water bank, spatial albedo and landscape shape, dPC, and windward width of each green space.

### 3.2. Structural Spatial Variables Affecting Cooling Effects from the Green Space Network

#### 3.2.1. Distance from Water Body (Dwb)

**Figure 8:**
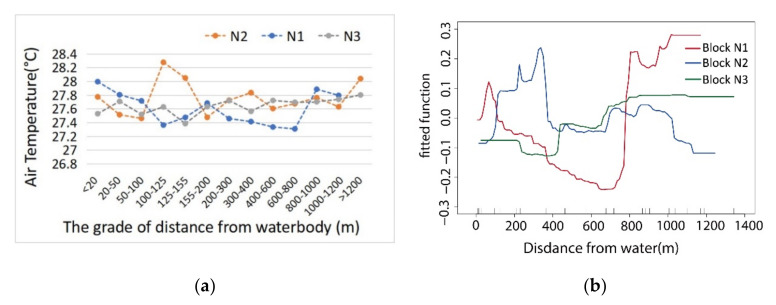
(**a**) Comparative marginal effect (ME) curve analysis via boosted regression trees (BRT) regression between the distance from water body (Dwb) and T value of green space; (**b**) correlation comparison between the DWb and T values of the green space.

As shown by the analysis of the ME change between the distance from the water body and the temperature of the green network (Figure 8a), the farther block N1 was from the water body, the more the ME of the water body to the inner T value of the green space decreased. The ME of the water body disappeared when this distance reached 700 m. Due to the narrow shape of the waterfront green belt, the waterfront’s cooling function was weak. The trends and fluctuations of the curve also indicated that the cooling function of the water body to the green network decreased steadily with an increase in distance from the riverbank. In block N2, the large ME of water on the internal temperature of the green space appeared at a distance range of 0–400 m from the water body and then decreased significantly. Within a 0–400 m range, the E–W orientation patterns of the green corridor in this area were seriously disturbed by the space of the building forms, resulting in a great fluctuation of the ME curve. The building layout separated the cold air circulation, and the curve change was low after 400 m. In block N3, the influence of green space along the bank line combined with the water body was higher than that of the other two blocks, which was close to 890 m with the largest ME. Similar to block N2, the widths of the green belt along the river bank in the two blocks were all approximately 95 m. As the eastern green space was divided by a construction site, in the range of 0–400 m from the water body, the cooling effect of the green space was obviously lower than that of block N2. However, due to the S–N green corridor distributed in block N3, the impact of distance from the water body on the cooling effect occurred relatively farther away and was more stable.

The water body located in the south of the block area had a significant cooling effect on the green space network within a certain distance and presented a negative correlation (Figure 8b). A comparison of the cooling effects of the three blocks showed that, in terms of cooling intensity, the green space pattern of block N1 was the best for the synergistic cooling effect (SCE) of the water body and green space. The cooling effect of the main corridor with a southeast to northwest orientation was the most stable and had the largest cooling range. If a waterfront with a wide riverside green space were planned, its cooling effect would be the strongest among the three study areas. In terms of the influence range of temperature reduction, the S–N green corridor played an active role in transmitting cold air from the water body and riverside green space, and the wide riverside green space was set to enhance the influence range of the cooling effect. Although block N2 had a wide riparian green space, due to the east–west orientation of the green space corridor, the cooling effect of the internal green space network was the worst.

#### 3.2.2. The dPC (Decreased Probability Connectivity) Variable

**Figure 9:**
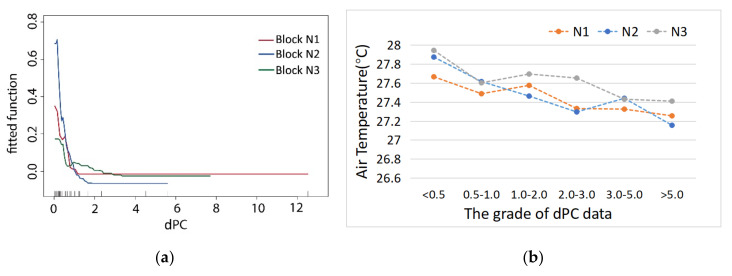
(**a**) Comparative ME curve analysis via BRT regression between the decrease in probability of connectivity (dPC) values and T values of the green space; (**b**) correlation comparison between the dPC values and T values of the green space.

According to the regression analysis, the correlation between the dPC index (used to describe the green space connectivity), and the T values, the connectivity of the green space with the landscape index has an obvious impact on the cooling effect within different blocks (Figure 9a). The dPC of most green spaces in block N1 was low, and the air temperature changed little with an increase in dPC values. The ME of the dPC for air temperature tended to be straight and sharp when the connectivity reached 1.5; in block N2, with an increase in connectivity, the variation range of the air temperature values was broader. The fluctuation of air temperature in the green space was at a middle level. The ME of green space dPC on the T value tended to be straight when the dPC values reached 1.8. The green space of block N3 was dominated by S–N corridors, as well as some green patches. The integrity degree of the green space network was lower than that of block N2, and the air temperature values corresponding to the dPC values of block N3 fluctuated more significantly. The ME of dPC on the cooling effect decreased and tended to become stable when the dPC values reached 3.6.

With an increase in the dPC of the three blocks, the internal T value of green space generally became correspondingly lower (Figure 9b). The results showed that the dPC of the green space network of block N1 was the best, where the fluctuation change of the T values inside the green space network was relatively small, and the total internal T value was the lowest. The internal T value fluctuation of the green corridor in block N2 was small and had multitudinous narrow east–west green corridors parallel to the riverbank. The dPC degree of the S–N green corridors in block N3 was less than that in block N1, and the correlation characteristics between the T values and the dPC values of the green space were basically consistent with those of block N1. As its interior and edges were affected by different types of vegetation coverage, water bodies, and surrounding environments, the internal T value fluctuation of the green corridor of the green space corridor was the largest, presenting a large inclination for the total correlation tendency. Therefore, the larger the connectivity values of the green space were, the better the air flow exchange between the green spaces was. In this situation, the cool island was also more effective, with the inner T values of the green space being more even and stable.

#### 3.2.3. Green Corridor Orientation

**Figure 10:**
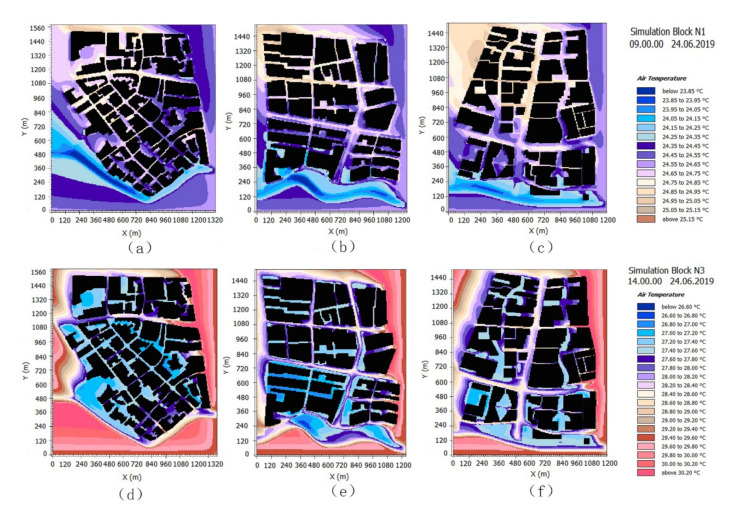
The spatial distribution of the T values in the three blocks at a height of 1.5 m. (**a**) Block N1, at 9:00 a.m.; (**b**) Block N2, at 9:00 a.m.; (**c**) Block N3, at 9:00 a.m.; (**d**) Block N1, at 14:00; (**e**) Block N2, at 14:00; (**f**) Block N3, at 14:00.

The orientation of green corridors is a categorical variable, so its values had a relatively small impact on the regression analysis in this research. However, the orientation factor is related to changes in network’s openness to the wind direction, connectivity with water bodies, and other landscape pattern index factors. From the distribution patterns of the simulation results, we clearly found that the degree of inclination between the green spaces and water bodies produced obvious correlation characteristics among the T value distributions of the green space network in each study area (Figure 10).

**Figure 11:**
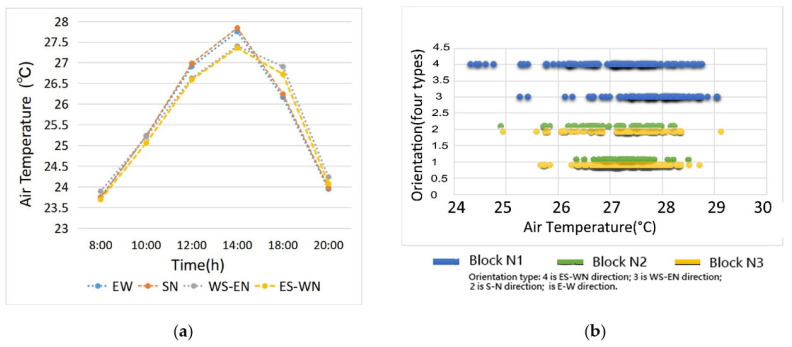
(**a**) Correlation comparison of the variation tendency between the orientation factors and T values of the green space during different hours and (**b**) a scatter diagram analysis of their correlation between the average T values and the orientation types of green corridors at 14:00 a.m.

The change tendency of the correlation between the T values and four orientation types in the three blocks are provided in Figure 11a. Before 15:00, the T values from low to high were SE–NW, SW–NE, E–W, and S–N. The results show that the green corridors with a SE–NW orientation had the lowest T values, indicating that the downwind areas have a better cooling effect. The low T values in the green corridors with southwest to northeast orientations were caused by the air circulation inside the spatial network, and the E–W orientation type was lower than the S–N orientation type, which highlighted the cooling effect of the surrounding building shadow. After 15:00, the T values of the E–W and S–N orientation types were better than those of the SW–NE and SE–NW types. This situation was observed in block N1, which is mainly composed of SW–NE and SE–NW green corridors. However, the width of the green corridors was narrow, and the air exchange was relatively slow.

According to the scatter diagram between the corridor orientation and the T values of the green corridors, which were obtained from the simulation data at 14:00 (Figure 11b), the green corridors with a SE–NW orientation in block N1 (where ordinate 4 was located) were distributed in a lower T value range. The T values of the green corridors with the S–N orientation (ordinate 2 position) in block N2 and block N3 were obviously lower than those of the E–W orientation (ordinate 1 position). This result (the cooling effect with S–N orientation > E–W orientation) of the scatter distribution in Figure 11b at 14:00 was inconsistent with the tendency in different hours of Figure 11a. This could be explained by the number of large riparian green spaces was classified into E–W orientation in block N2 and block N3, which led to a large number of E–W orientation green spaces with low-temperature values in the two blocks, producing results for the average T value of the E–W orientation type that were slightly better than those of the S–N orientation type. In comparison, the cooling effect of block N3 was better than that of block N2.

According to the above analysis results, in the whole green space network construction, the orientation of green corridors had an important impact on cooling. The analysis results show that in the summer, when the southeast monsoon prevails, the order of cooling effects from high to low is SE–NW > SW–NE and S–N > E–W.

#### 3.2.4. MSPA Types

**Figure 12:**
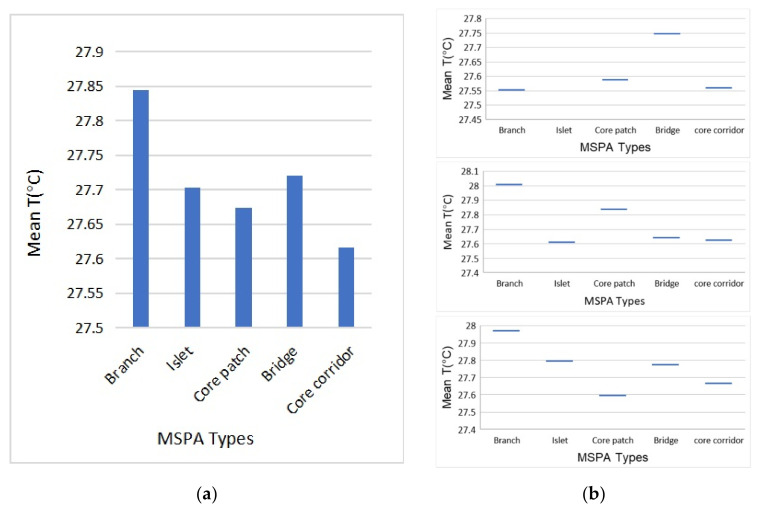
(**a**) Mean temperature variation of different morphological spatial pattern analysis (MSPA) types of green space from the holistic patterns and (**b**) the individual correlation analysis between the MSPA types and the mean temperature of green space in each of the three blocks.

The green space in the three blocks was divided into branch, islet, core patch, bridge, and core corridor types based on the MSPA analysis. All the classified green spaces were analysed via mean temperature correlation analysis (Mean T) (Figure 12a). The mean T value of the core corridor type was the lowest, which indicated that the corridor continuity experienced the best cooling effect in the network, followed by the core patch, which indicates the cooling impact at the green space scale. The branch type of green space had the highest mean T value, as the linear green space had no certain width scale, and its thermal environment was the worst. The bridge type was the spatial form caused by continuity interruption of the green corridors, and the integrity of the green corridors was damaged. The thermal environment of this type was also poor.

By comparing and analysing the spatial forms of the green space network of the three blocks (Figure 12b), we determined that the integrity of the green network in block N1 was relatively better, with no observed islet type of green space. The main structure was the green corridor type, which was the main low-temperature distribution area in the green space network. There were large quantities of the branch type and bridge type with narrow widths, and their cooling effect was not good. This result indicates that although connectivity played the most important role in the cooling process, the fragmentation and insufficient scale of green space could not produce a good cooling effect. The green space network of block N2 was highly fragmented, and there were separate islet types. The types of core corridors in block N2 also had the strongest cooling effect. The island type had larger green space patches, which could also produce a better cooling effect. In block N3, the network structure of the green space was relatively integrated, and there were large core patch types surrounded by the building groups. Large core patches had the best cooling effect in the block. The number and scale of the other MSPA types of green space were low, and their cooling effect was very poor.

In terms of the above analysis, the core corridor type with a large width played the most important role in the cooling effect for the overall green network. When the green space network is connected, the cooling of all types of green space is in its optimal state. When the network connectivity is insufficient, large green patches can also produce a better cooling effect.

### 3.3. Cooling Impact Analysis of the Spatial Morphology Variables

#### The Variables of the Surface Coverage Morphology and Ambient Factors

For two-dimensional plane formation, the surface coverage morphology and ambient factors are two important elements affecting the distribution of the T values. Among the spatial morphological factors that affect the cooling effects of green space, the variables of VS_Albedo_ and T_surface_ were used to reflect these two factors. In the BRT model analysis, these two factors were the most important variables to form the dynamic correlation of the simulation T values. In the three study areas, these two variables had different degrees of influence.

**Figure 13:**
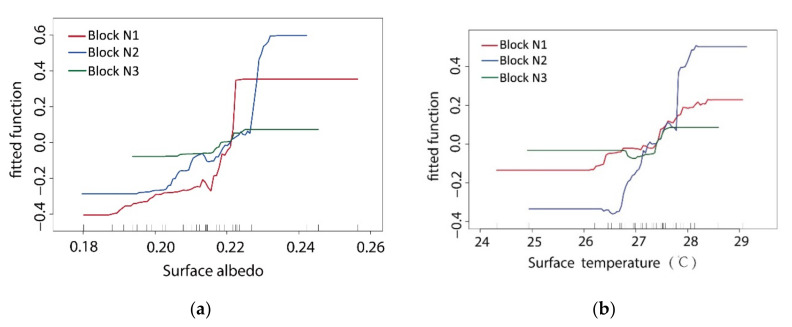
Comparative ME curve analysis via BRT regression (**a**) between the VS_Albedo_ and T value of green space and (**b**) between the T_surface_ and T value of green space.

The distribution of VS_Albedo_ basically relates to the vegetation coverage, the aggregation degree of the green space pattern, and the adjacent surrounding spatial pattern. In this study, the configuration mode of vegetation coverage factors was set with the same status, and VS_Albedo_ was mainly affected by the agglomeration degree of the green space pattern and the surrounding spatial pattern. Based on an analysis of the ME curve relationship between VS_Albedo_ and the T value (Figure 13a), the gradient of the ME curve of block N1 was the largest. Comparing the three blocks, increasing the albedo of the green space in block N1 obviously decreased the T value. This increase in the agglomeration degree of the green spatial pattern and decrease in the surrounding spatial pattern impacts all show that if the widths of the green corridors in the block are increased, a more stable low-temperature internal environment emerges in the internal green space. In one sense, this result indicates that the scale of green corridors became a limiting factor for this effect. The gradient values of the ME curve of block N2 and block N3 were consistent as a whole, but in block N3, the change range of the T value was very small. Specifically, the cooling change inside the green space of block N3 was more stable than that of block N2.

**Figure 14:**
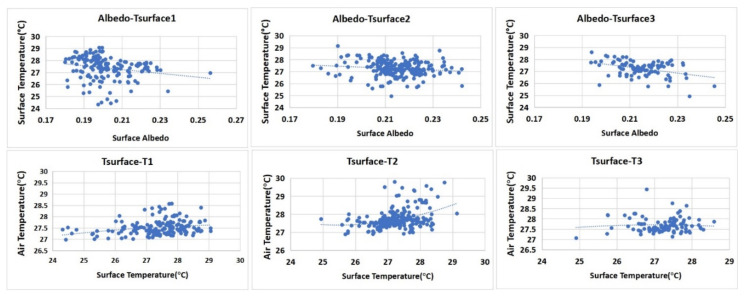
Upper map: scatter diagram of the correlation analysis between VS_Albedo_ and the surface; lower map: scatter diagram of the correlation analysis between the T_surface_ and T of the green space in the three blocks.

The variable of T_surface_ was affected by the albedo status from the surface coverage and was also directly related to the heat absorption properties of different ambient materials adjacent to the space (the water body in the adjacent water area and high albedo building material). Through an analysis of the ME curve between the T_surface_ and T values (Figure 13b), it can be found that the gradient of the ME curve of block N2 was the largest. As its VS_Albedo_ change was consistent with that of block N3 overall (Figure 14 Upper map), a difference in the ME of block N2 should have produced an obvious difference in the spatial patterns of the surrounding buildings. In block N2, the green corridors are mostly surrounded by E–W buildings, and at the end of the branch, a more closed node space is distributed. These two spatial patterns produced the maximum ME of the T_surface_ change due to the different surrounding surface materials. The gradients of the ME curve of T_surface_ in block N1 and block N3 were stable; the changes in T_surface_ in the internal green spaces of the two blocks were small (Figure 14 Lower map), and the correlations with the T values in the two blocks were relatively weak.

There is a negative correlation between VS_Albedo_ and T_surface_. The heating effect of the atmosphere from longwave radiation of T_surface_ was much higher than that directly generated by solar radiation. Thus, there was a negative correlation between T_surface_ and the T value. The higher the VS_Albedo_ value was, the lower the temperature was. However, the interference of other ambient factors that affect the thermal environment of the green space weakened this direct correlation. The common characteristics of T_surface_ distribution were as follows: (1) The area adjacent to the water body was affected by the cool source of the water body, and the T_surface_ was a low value area; (2) the green space enclosed by the boundary of the building group was the high-value area of T_surface_; and (3) there was a high-temperature area in the adjacent road intersection area. In general, the spatial pattern distribution of the VS_Albedo_ index and the T value distribution map of the simulation results at the 1.5 m ground level showed a significant correlation in their spatial patterns.

### 3.4. Sky View Factor (SVF)

**Figure 15:**
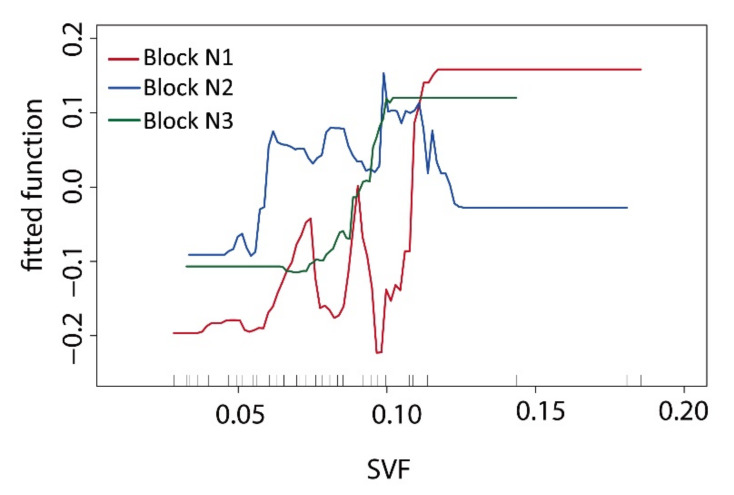
Comparative ME curve analysis via BRT regression between the SVF values and T value of the green space in three blocks.

The SVF can be defined as the ratio of the radiation from the sky not intercepted by objects to the maximum radiation from the sky. SVF is an important index describing the three-dimensional configuration of green space [66]. In this study, the proportion of trees in the green space was large, which made the SVF values in the green space generally low. The ME curve relationship between the SVF data and T values in the green space (Figure 15) showed that the curve of block N1 fluctuated greatly. The internal building groups separated their space into fragmented shapes, and the cooling effect fluctuated significantly in green spaces with changes in the SVF values. The curve gradient of the ME in block N3 was consistent with that in block N1. The SVF data of the green space also had excellent correlation with the T values of the green space in block N3. The edge shape of the green space enclosed with regular buildings in block N2 was relatively simple, and the SVF values changed very little. Its influence on green space was relatively stable, and its correlation degree was very low.

**Figure 16:**
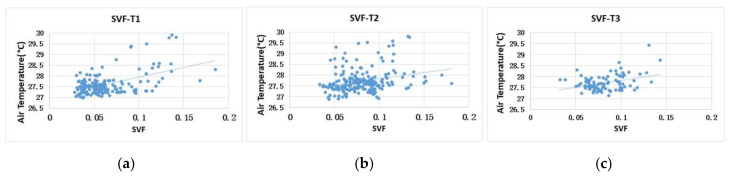
Scatter diagram of the correlation analysis between the SVF values and the T value of the green space in the three blocks. (**a**) Block N1; (**b**) Block N2; (**c**) Block N1.

The correlation between SVF and temperature in the green space was analysed to obtain the interactive characteristics (Figure 16). The SVF data and spatial T values were found to be positively correlated. This result is consistent with existing research conclusions [34,50]. As a three-dimensional spatial factor, SVF was very important for the T value distribution of green space in our research. The canopy coverage of trees and the openness degree of the whole spatial network determined the cooling effectiveness of the SVF index. The inner space with better tree coverage had a strong cooling effect; in the green space network, under a high density of trees, a properly open SVF could be conducive to forming many low T value zones.

#### Green Corridor Width (GWd)

The scale of green space is an important spatial factor for describing the green space’s characteristics. The GWd not only reflects the influence of green quantity on the cooling effect but also includes the effect of ventilation corridors in creating a cooling effect. The GWd is not only a quantitative measurement of that space but also reflects its spatial pattern.

**Figure 17:**
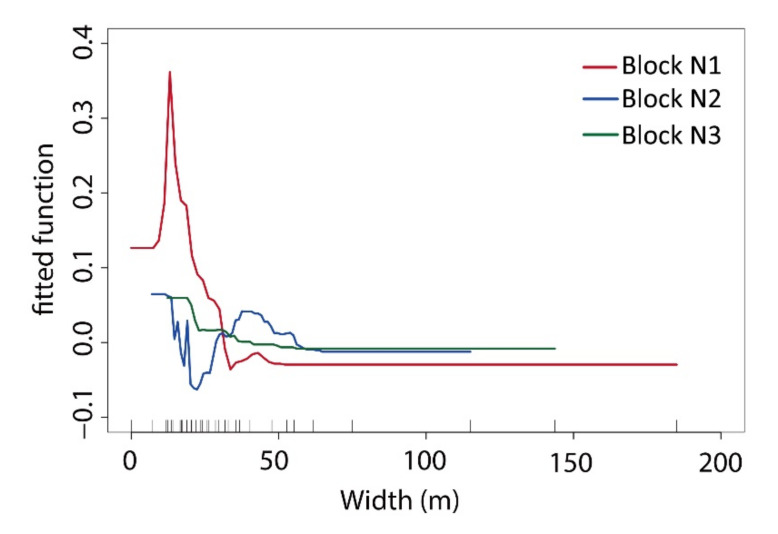
Comparative ME curve analysis via BRT regression between the green corridor width and T value in the three blocks.

Based on an analysis of the ME curve of green space between the width data and T values (Figure 17), the curve gradient of the ME in block N1 was larger than block N2 and block N3. The maximum width impacting the cooling effect in block N1 was 50 m, that in block N2 was 55 m, and that in block N3 was 58 m. When the GWd in block N1 was 20 metres, the block’s cooling ME reached its maximum. The GWd varied greatly, resulting in obvious fluctuations in the cooling effect. When the GWd in block N2 was between 25 and 40 m, the ME of the cooling effect continued to increase in width. Beyond 55 metres, the ME of green space cooling began to stabilize. When the GWd was less than 25 metres, its cooling effect was especially affected by the surrounding ambient environment, but the width had no regular impact on the T value changes of the inner green space. The change range of the ME was the smallest because most green space corridors were already large enough in the block.

**Figure 18:**
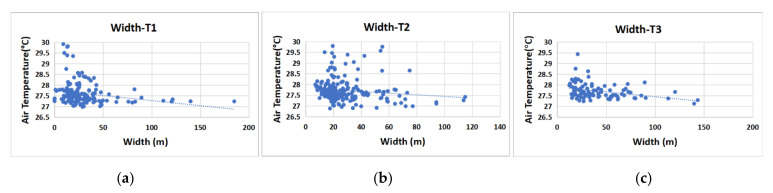
Scatter diagram of the correlation analysis between the green corridor width and the T value in the three blocks. (**a**) Block N1; (**b**) Block N2; (**c**) Block N1.

The cooling effect of green space in the three blocks became stronger with an increase in width (Figure 18). Due to the different landscape patterns of green spaces, the wide range of influences on the cooling effect of green space was also different in the three blocks. In block N1, the GWd increase created an obvious cooling effect. However, the widths of block N1′s green corridors were largely in a lower range, which affected the overall cooling efficiency of the green space. In block N2, the green corridors with an E–W orientation were relatively narrow, and the cooling changes for this kind of width were very small. The green space of block N2 was greatly disturbed by buildings and other ambient factors, and the connectivity of this block’s green corridors was also insufficient, creating a situation where increasing the GWd had little effect on the overall cooling efficiency of the green space. In block N3, the GWd was larger, and the cooling efficiency became gradually flat with an increase in width.

As mentioned above, when the width of the green corridor was between 0 and 55 m, the cooling effect of the green space was obvious. When the GWd was greater than 55 m, the ME of the cooling reached its maximum, and the influence tended to be stable. It is thus very important to keep the GWd at 20 m to promote a cooling effect.

## 4. Discussion

### 4.1. Implications for Urban Climatic Adaptive Design

Waterfront areas are an ideal human settlement environment. Since Shanghai is a city with hot summers and a large number of river networks, a reasonable layout study of microclimate effects in waterfront areas can help provide prospects for developing a more thermally comfortable space. In this study, we analysed the cooling effects of three typical waterfront green space networks with different spatial structures to identify the impact of cooling effect factors from green space network indices and morphology indices. Through a comparative study and statistical analyses, the green network of waterfront blocks with good cooling effects had the following structural and morphological characteristics:

(1) Optimal spatial composition between blue and green space for maximized SCE.

There were great differences in the SCEs with different spatial compositions of the blue–green space model in the three study areas. It was found that the cooling effect of the water green cold island reached the first turning point at 100 m in block N1, with a relatively narrow waterfront green belt about 35 m wide. Compared with block N1, the waterfront green belts in block N2 and block N3 were wider, with a width of 90–100 m, the distances that influenced the cooling effect were all significantly larger than those of block N1. The characteristic inflection point of the cooling effect in block N2 was about 400 m, while that of N3 was between 500 and 600 m (Figure 9a). It can be seen that the width and integrity of the riparian green belt had a significant impact on the range of the SCE. While the widths of the water bodies and riparian green spaces were similar, the SE–NW and S–N orientations of the green corridors that were consistent with the direction of the summer monsoon formed a cold air diffusion path from the southern cold source, and the maximum influence distances of cooling synergy were strengthened by the waterfront green corridor system with conduction and circulation functions.

In terms of the relationship between the green spatial pattern and the water body distribution, as well as the synergistic effects, by distributing the green corridors, the wide riparian green belt and cold air diffusion path became the optimal blue–green spatial composition pattern, offering the largest cooling impact distance.

(2) Practical structural patterns of the waterfront green space network for holistic cooling effects.

The green grid structure of the waterfront area offered the best spatial composition mode for green space cooling, based on the comparative study. A green space system with an SE–NW or S–N orientation of its green corridors was the main structural framework. The main spatial structures of the green corridors featuring orientations consistent with the direction of the summer monsoon could help cooling air reach the unobstructed internal space of the green corridors during summer. Under the dominant green corridor structure, the corridor with E–W connectivity also played an important role in internal air circulation and exchange; the E–W corridor was generally enclosed in the built-up area, and its architectural interface produced a street valley effect and shielding effect, further strengthening the internal air circulation.

The cooling effect was better for green space networks with high connectivity and sufficient width. The spatial composition had different landscape shapes and ecological functional types, and each green space type should increase its connectivity to the green structure framework. The denser the green corridor was, the higher the aggregation degree of the green pattern was, and the better the connectivity with the adjacent green space became. The other restricting factor of the green grid network for the cooling effect was the width factor of the green corridor. The presence of a green corridor with a width of 20–50 m is an important factor to ensure the cooling intensity of the green space, although the cooling effect of this width was uncertain in different local regions.

(3) Complex influence of three-dimensional morphology and the ambient interface of the green space network.

The three-dimensional distribution of vegetation and the three-dimensional spatial characteristics of the surrounding interface are the most important factors affecting the variation of T values in green space. 3D indices play a more important role than 2D indices in studying the T_surface_ values of different urban geometric shapes [67]. In terms of the three-dimensional openness of the green space network, the three-dimensional shape factor is SVF. The vegetation SVF variable related to the reflectance and heat absorption of spatial characteristics have important effects on the spatial variation of the T value in green space. Strong, weak, and no significant relationships between SVF and UHI have been variously reported, even for the same urban areas [68]. In our study, the smaller the sky width factor of the green space is, the better the overall cooling effect of the green space is—e.g., the overall cooling effect of the green space is better when the vegetation coverage is good in the summer. However, the configuration structure of compound vegetation also needs to be open to a certain extent, which will facilitate the formation of air flow in green space. The SVF and T values have a weak correlation in some special data points in the study area, with a complex influence on the cooling effect.

The green spaces with higher surface albedo have more stable surface temperature change. The surface albedo of green space was an important factor producing the different spatial partitions of air temperature in the internal green space. The effect of VS_Albedo_ on T value is also complex. The changes of surface albedo were affected by plant growth, soil surface water content, and ambient environment. There is a coexistence of positive and negative correlation between surface albedo and T value change [69]. In our study, the relationship had negative correlation tendency between VS_Albedo_ and T value, which is consistent with most of the existing research results in hot and humid location. Thus, the spatial characteristics of high reflectance material and low absorptivity colour of the green space ambient are the preferred space design form for the cooling effect.

### 4.2. Differences from Previous Studies

The cooling effects of blue-green collaborative spatial patterns emerge from a very complex and comprehensive ecological process. Previous studies showed that the cooling effect of the blue-green space depends on the size, shape, type, density, connectivity, and complexity (components and configuration) of the blue-green space, as well as the greenness of the green vegetation [5,44]. On the other hand, many researchers have realized that quantifying the threshold sizes of different landscape types, including the effects of landscape composition and configuration, is essential for decision-makers to obtain the maximum cooling efficiency of blue–green space and to develop actionable climate adaption plans [5]. However, most existing studies have discussed the cooling effects of urban green space only at a macro scale. Moreover, previous studies mainly addressed green space and water bodies from the perspective of separation, while few studies considered the synergistic cooling effects of blue and green spaces [1].

Researching the synergetic effects of blue-green spaces in actual built-up areas is very complex. Such studies need to integrate multiple elements and multi-dimensional spatial factors to ensure a comprehensive model quantification and impact correlation analysis. In this study, a quantitative description of the multi-dimensional spatial, structural, and morphological factors of urban mesoscale built-up areas was adopted, and the BRT machine learning method was used to explore the influence importance degree. Moreover, the threshold value of efficiency (TVoE), or impact tendency, of each index factor was provided to illustrate the distribution characteristics of each factor’s cooling effects. This study is in line with the constraint factors of planning and layouts and can provide guidance based on specific layout factors for practical local construction.

### 4.3. Limitations of This Study

The research was limited to a correlation analysis between the spatial structure and composition factors of the green space and temperature distribution. The diurnal and seasonal variations in the synergistic effects of the WCI and UGCI were not analysed. A Comparison of different climatic regions and seasons, as well as day and night data, should also be used in the future to make the results more comprehensive. Likewise, more variables, such as the types of vegetation, the heights of buildings, wind speed, etc., should be considered in subsequent studies, as this research only considered a general decrease in the T value within the typical spatial structure of a green space network. A further comparative study on the spatial configuration of green space network with different model characteristics, or different types of Local Climate Zone (LCZ) in a wider regions or locations, would eventually provide the main implications of their performance variations on the urban design.

## 5. Conclusions

Based on the three-dimensional numerical simulation method, the cool island effects of the green space systems in three built-up blocks of Shanghai by Suzhou Creek were compared and analysed. Block N1 had the best overall cooling effect, while block N3 took second place, and block N2 had the worst cooling effect. The results showed that the effect of the cool island in block N1 was stable; the cooling efficiency of block N2 fluctuated greatly, and the cooling effect of block N3 was greater than that of block N2 during the summer.

In the green space description index system of the waterfront area, distance from a water body, green corridor orientation, green space connectivity, MSPA type, spatial pattern, three-dimensional morphological factors, and scale factors were the restrictive factors that impacted the cooling effect. The main influencing index was selected for specific analyses. The riparian green space with better integrity and a larger area had a better SCE, and the distance from a water body had a negative correlation with the T value of the green space. Under the synergistic effect of water bodies and green space in our research, the cooling effect range was 800–1000 m, and the connectivity of the green space had a negative correlation with the T value of the green space. The cooling effect of the corridor with greater connectivity was better than that of the green patch. There was a negative correlation between VS_Albdo_ and the T value. This study showed that there is a consistent correlation between the VS_Albdo_ distribution and the internal differentiation of the green space at the microscale; moreover, the SVF was positively correlated with the T value. The green space width was also positively correlated with the T value of the green space. When the width of the green space was 20–25 m, the ME of cooling was the largest; when the width was greater than 55 m, the cooling effect tended to be stable.

The microclimates of three typical pattern cases in three blocks in Shanghai were simulated via ENVI-met. The composition elements of each green space influencing the microclimate were analysed from two spatial levels: the green space network and every green space. The conclusions of the ME analysis and threshold values of the BRT model will be useful for urban planners who are developing strategies to improve the outdoor microclimate. Shanghai is in a mature stage of urbanization development, and a variety of structures featuring waterfront built-up areas need to be updated with more precise features. The structures of green space networks like block N1 should improve the width and openness of their green spaces. The spatial structure of green space networks like block N2 need to increase the connectivity of their green spaces and the widths of their green corridors to improve their microclimate conditions. Networks like block N3 are greatly affected by water bodies and the local surrounding built-up environment. Increasing the connectivity from east to west with green corridors and properly improving the local openness of green spaces will further enhance the overall cooling effect in such areas.

As a weakness, this study’s choice of green spatial patterns could have been better diversified, and further in-depth studies on the roles of relevant factors need to be performed. In general, the BRT model yielded a comprehensive comparative analysis of all factors in the built-up area. This research method is worthy of being applied to more medium-sized quantitative studies, and a combination of multispectral and multiresolution remote sensing images and GIS analysis could be used in future research processes to ensure a more in-depth comparative analysis.

## Figures and Tables

**Table 1:** Selection of the multidimensional spatial variables to describe the green space network based on the cooling effect.

First Level Variables	Second Level Variables	Definition and Description	Values Assignment	Adapted from
Network structure variables	DWb	The distance between the geometric centre of green space and the riverbank representing the influence of the water body on the cooling effect of the green space.	Calculating the distance value via proximity analysis in ArcGIS 10.4	[1,19]
	Orientation	The trend of the long side of green space for especially reflecting the cooling influence from the green space corridor and the wind direction inclination angle.	Categorical variable; value 1 = E–W orientation; 2 = S–N; 3 = SW–NE; 4 = SE–NW	[16,34]
	MSPA types	The mathematical morphology types by the Morphological spatial pattern analysis (MSPA). The importance of connectivity categories is extracted.	Categorical variable; value 1 = branch; 2 = islet; 3 = core patch; 4 = bridge; 5 = core corridor	[35,36,37]
	dPC	The connectivity index characterizing the contribution of each green space in the connectivity to the overall green space network.	Using the data from landscape connectivity evaluation software, Conefor Sensinode 2.6 (by Santiago Saura and Josep Torné, at the Polytechnic University of Madrid and the University of Lleida, Spain)	[38,39,40]
	GR	The ratio of the sum of green space area within the scope of land use to the total land use. The values were almost equal in three case study areas.	Presetting to a control variable and excluding from correlation factor analysis	[26,41,42]
	VSt	Three-dimensional spatial configuration of vegetation in each green space. The proportions of the vegetation configuration were set the same, the ratio of the number of trees and grass area is 1:30 plant/m^2^.	Presetting a control variable and excluding it from correlation factor analysis	[27,43]
Spatial morphology variables	GA	The surface area that the green space occupies	Calculating the distance value via spatial analysis in ArcGIS 10.4	[44,45,46,47]
	GWd	The cross-sectional width of the green corridor that is perpendicular to the ventilation direction of the green space.	Calculating the distance value by spatial analysis in ArcGIS 10.4	[48,49]
	P/C pattern	Landscape essential factor types of the patch–corridor–matrix model of landscape pattern, for comparison landscape composition of the microclimatic differences.	Categorical variable; value 1 = patch, 2 = corridor	[15,26]
	LSI	The shape complexity index measured by calculating the deviation degree between the shape of the green space parcel and its square of the same area.	Calculating by Patch analyst 5.1 package in ArcGIS 10.4	[15,44]
	SVF	The ratio of sky hemisphere visible from the ground (not obstructed by buildings, terrain or trees), three-dimensional morphological parameters.	Simulation data of the surface conditions via ENVI-met 4.4.	[28,42,50]
	VS_Albedo_	The ratio of the surface reflection flux to the incident solar radiation flux on the surface of green space.	Simulation data of the VS_Albedo_ via ENVI-met 4.4	[6,29]
	T_surface_	The ground surface temperature of green space, representing the impact from the three-dimensional spatial difference of green space and ambient factors.	Simulation data of the T surface via ENVI-met 4.4.	[6,29]

DWb: Distance from water body; GR: Green space ratio; VSt: Vegetation structure; dPC: The decrease probability connectivity; MSPA: Morphological spatial pattern analysis; LSI: Landscape Shape Index; P/C pattern: Patch/Corridor pattern; T_surface_: Surface temperature (°C); VS_Albedo_: Vegetation surface albedo; SVF: Sky view factor; T: Air temperature (°C); ΔT: The difference between T and the mean T of the entire study area; CV-T: Coefficient of variation of air temperature.

**Table 2:** Initial input values of weather parameters for the simulation model.

Input Parameters	T (℃)	Wind Orientation	WS (M/S)	Humidity (%)	Roughness
Value	24.51	135 °	5.53	66.46	0.01

**Table 3:** Parameter settings of spatial models in three study zones.

Simulation Parameter Settings	Area		
	Block N1	Block N2	Block N3
Number of grids (x, y, z)	233, 263, 45	206, 256, 45	198, 253, 45
Size of grid cell (m) (dx, dy, and dz)	6, 6, 3	6, 6, 3	6, 6, 3
The material for nesting grids	Soil A: Loamy Soil Soil B: Loamy Soil	Soil A: Loamy Soil Soil B: Loamy Soil	Soil A: Loamy Soil Soil B: Loamy Soil
Default wall material	moderate insulation	moderate insulation	moderate insulation
Default roof material	moderate insulation	moderate insulation	moderate insulation
Street material	Loamy Soil	Loamy Soil	Loamy Soil

**Table 4:** Relative importance of the green space indices for each model.

Spatial Variables of Morphological Indices	Relative Importance of Predictor Variables		
	Block 1	Block 2	Block 3
DWb	18.11%	10.79%	14.78%
VS_Albedo_	21.89%	16.72%	10.00%
T_surface_	6.61%	18.45%	9.77%
dPC	4.09%	19.34%	14.52%
MSPA types	1.46%	5.24%	5.11%
SVF	22.18%	10.53%	22.69%
MSI	5.29%	6.60%	8.56%
GWd	10.67%	3.69%	5.48%
VSt	2.78%	1.29%	3.28%
GA	3.50%	7.06%	5.47%
Orientation	3.23%	0.24%	0.33%
P/C pattern	0.18%	0.06%	0.00%

## Abbreviations

Synergistic cooling effect; UCI: Urban cool	SCE
Urban heat island	UHI
Water cool island	WCI
Urban green space cool island	UGCI
Air temperature (°C)	T
Surface temperature (°C)	T_surface_
The difference between T and the mean T of the entire study area	ΔT
Coefficient of variation	CV
Coefficient of variation of air temperature	CV-T
Boosted regression trees	BRT
Marginal effect	ME
threshold value of efficiency	TVoE
Block no. 1/no. 2/no.	Block N1/N2/N3
East-west orientation	E–W
South-north orientation	S–N
Southwest-northeast orientation	WS–EN
Southeast-northwest orientation	ES–WN
Morphological spatial pattern analysis	MSPA
Probability of connectivity	PC
The decrease probability connectivity	dPC
Distance from water body	DWb
Green corridor width	GWd
Landscape Shape Index	LSI
Green space ratio	GR
Green space area	GA
Sky view factor	SVF
Vegetation structure	VSt
Vegetation surface albedo	VS_Albedo_
Patch/Corridor pattern	P/C pattern
Local Climate Zone	LCZ
